# Clitoromegaly Secondary to Exogenous Androgen Exposure From Paternal Skin to Skin Transfer

**DOI:** 10.7759/cureus.31101

**Published:** 2022-11-04

**Authors:** Seth J Deskins, Felistia Crowder, Elizabeth Proenza, Brian Ely

**Affiliations:** 1 Internal Medicine-Pediatrics, West Virginia University (WVU) School of Medicine, Morgantown, USA; 2 Pediatrics, West Virginia University (WVU) School of Medicine, Morgantown, USA; 3 Pediatric Endocrinology, West Virginia University (WVU) School of Medicine, Morgantown, USA

**Keywords:** transdermal testosterone, clitoromegaly, testosterone, endocrine pathology, pediatrics

## Abstract

Clitoromegaly is the abnormal enlargement of the clitoris. Its etiology is often divided into congenital and acquired causes, leading to a differential diagnosis that is quite broad. Workup often includes serum hormone testing, imaging studies, and sometimes an investigation into genetic and nonhormonal causes, which can be obtained from a detailed patient history.

Exposure to exogenous or endogenous androgens can directly stimulate and enlarge the clitoris, resulting in early virilization. Transdermal testosterone gel can be transferred from the skin of an adult to the skin of a child. Topical testosterone gel is an approved therapy for the treatment of hypoandrogenism in males. While it offers a convenient means of treatment, there is a concern about unintentional exposure to females and children from skin contact with the application site. Here, we report a case of an infant who presented to a pediatric endocrinology clinic for clitoromegaly that was possibly due to exogenous exposure to testosterone.

## Introduction

Despite the wide use of testosterone for multiple conditions, there are limited reports in the literature with regard to prenatal or postnatal androgen exposure leading to virilization in children [1,2]. Testosterone products are commonly used for the treatment of medical conditions including erectile dysfunction and reduced muscle mass [3], and increasingly, testosterone gel has been used in gender-affirming hormone therapy. It is estimated that around four million males in the United States alone suffer from hypogonadism, with nearly two million prescriptions ordered for testosterone replacement [3]. Secondary exposure to family members, through either direct exposure, as in this case due to father/parent-child interaction, or prenatal exposure via fetal placental transmission, can lead to virilization. We report a case of clitoromegaly secondary to inadvertent exposure to testosterone gel during normal parent-child interaction.

## Case presentation

A 13-month-old female presented to the pediatric endocrinology clinic with a two-month history of clitoromegaly. The patient’s parents noticed a diaper rash that appeared concurrently with the onset of clitoromegaly, which eventually resolved but with the persistence of clitoromegaly. The patient was born at 39 weeks without complications, with a normal newborn physical examination and development appropriate for age. Newborn screening results were normal, and no significant family history was reported. One month after symptom onset, the patient’s pediatrician had obtained screening results with electrolyte and 17-hydroxyprogesterone levels. These laboratory results were within the normal reference range including sodium of 137 mmol/L, potassium of 4.6 mmol/L, and 17-hydroxyprogesterone of 25 ng/dL. She was then referred to pediatric endocrinology for further evaluation.

**Figure 1:**
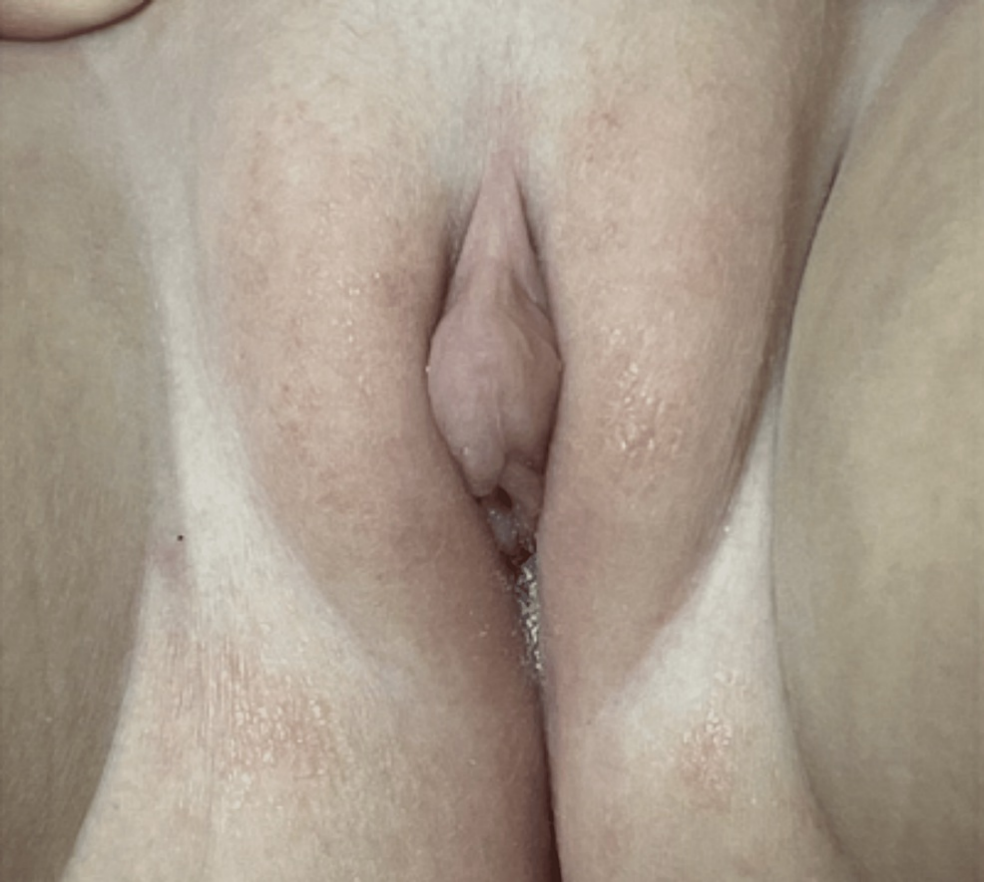
Prominence of the clitoris on initial examination

The patient was seen by endocrinology 20 days later, and physical examination was notable for a prominent clitoral hood with a normal vaginal opening with no posterior fusion (Figure 1). No breast buds or pubic hair were noted, but laboratory results were significant for a mild elevation in total testosterone (Table 1). Further history obtained from the family revealed that the patient’s father was applying testosterone gel to his shoulders and that the patient had a possible exposure. Upon awareness, the father switched from topical testosterone gel to intramuscular testosterone injections just after the initial visit to the endocrinology clinic.

**Table 1:** Laboratory data from initial encounter and at four-month follow-up

Initial visit	Four months later
Total testosterone: 25 ng/dL (normal: 8 ng/dL)	Free testosterone: 0.5 pg/mL (normal: 1.5 pg/mL)

**Figure 2:**
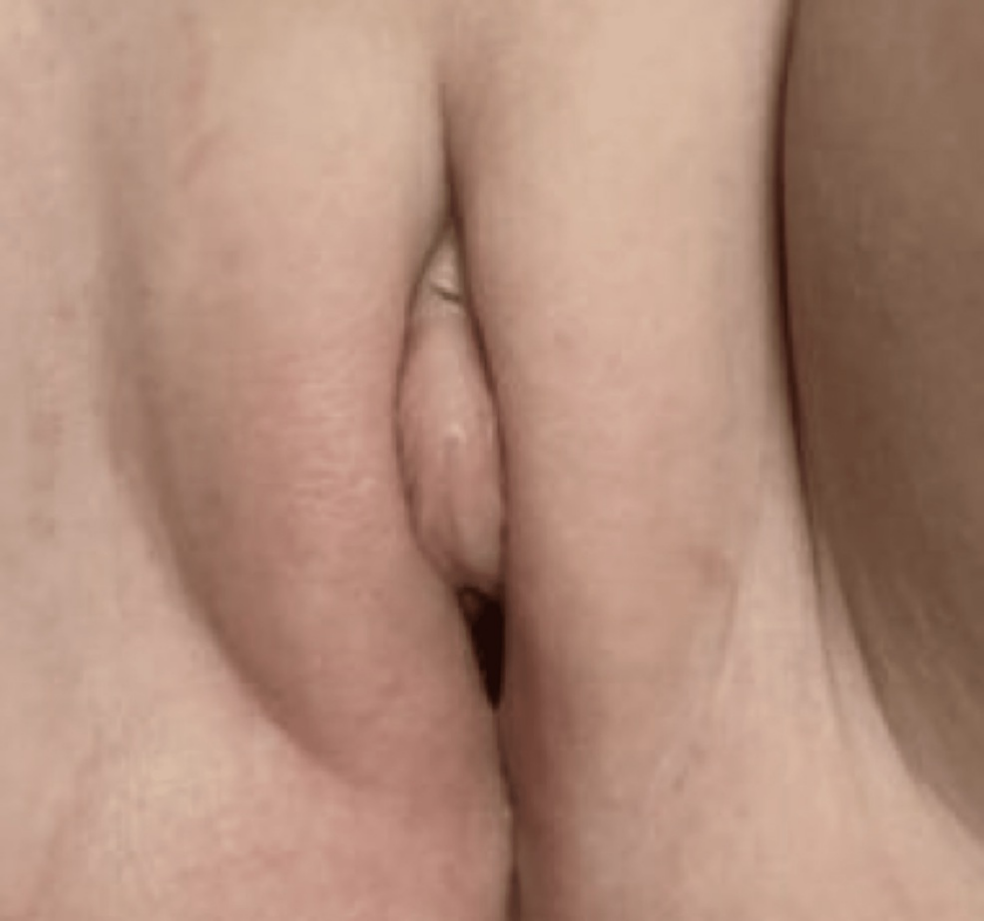
Decrease in clitoral prominence four months after the initial examination

Repeated laboratories four months later for the patient were all within normal ranges, including her free testosterone, dehydroepiandrosterone (DHEA), and androstenedione. Her clitoromegaly had improved, having no further features of virilization (Figure 2). Given the patient’s mild, initial elevations in androgen levels and its resolution on repeat testing, along with improvement in clitoromegaly after exposure removal, it was determined that virilization was a direct result of exposure to the father’s topical testosterone gel.

## Discussion

There are numerous case reports of clitoromegaly or the abnormal enlargement of the clitoris with or without clinical or biochemical hyperandrogenism [4]; less commonly reported is exogenous exposure from a male source. Undesired virilization is a known potential side effect of exposure to testosterone gel and was recognized by the Food and Drug Administration in May 2009 [3]. A boxed warning has been placed on the medication with directions for children to “avoid contact with unwashed or unclothed application sites in males using testosterone gel.” To that end, given the large number of males suffering from hypogonadism, physicians should be cognizant of incidental exposure [3]. Because the patient’s father denied skin to skin contact between his child and the application sites on his shoulders, it is suggested that the transmission primarily came from the residual testosterone gel left on his hands while he was holding, changing, and playing with his daughter.

We present this case to emphasize the adverse effects of topical testosterone and the potential side effects inherent to it. Not only can children develop enlarged genitalia, but also they can also present with any other feature of virilization [3]. There have been mixed reports about the reversal of virilization, with some cases showing resolution, while others show the persistence of clitoromegaly and advanced bone ages [3]. This report highlights the importance of attaining a thorough history when seeing patients with evidence of virilization. When evaluating a patient in the clinic with clitoromegaly, pertinent parental history of hypogonadism and current medications should be considered. All individuals using testosterone gel should be reminded to wash their hands after application or consider using gloves in applying the testosterone gel, particularly if a young child is in the home.

## Conclusions

Clitoromegaly is a commonly encountered diagnosis in pediatric endocrine clinics. Clitoral enlargement occurs due to either endogenous or exogenous androgen exposure. A recognized but less common cause is accidental exposure to testosterone gel through direct skin contact. This case demonstrates a patient referred for pediatric endocrinology consultation for clitoromegaly, with a further history revealing the cause to be exposure to paternal topical testosterone gel. With the removal of the offending agent, we saw a resolution of laboratory abnormalities and improvement of clitoromegaly without the need for more extensive laboratory and imaging studies.
